# Language development of premature children followed up in an outpatient clinic: a preliminary study

**DOI:** 10.1590/2317-1782/e20240284en

**Published:** 2026-04-10

**Authors:** Jonathan Gonçalves Rocha, Maisa Alves Teixeira, Andrezza Gonzalez Escarce, Thamara Suzi dos Santos, Denise Brandão de Oliveira e Britto, Stela Maris Aguiar Lemos

**Affiliations:** 1 Departamento de Fonoaudiologia, Faculdade de Medicina, Universidade Federal de Minas Gerais – UFMG - Belo Horizonte (MG), Brasil.; 2 Programa de Pós-graduação em Ciências Fonoaudiológicas, Faculdade de Medicina, Universidade Federal de Minas Gerais – UFMG - Belo Horizonte (MG), Brasil.

**Keywords:** Premature Birth, Child Language, Language Development, Child, Diagnosis

## Abstract

**Purpose:**

To verify the association between language development and sociodemographic, peri- and postnatal aspects of children with a history of prematurity followed up in an outpatient clinic.

**Methods:**

This was an observational, analytical, cross-sectional, and preliminary study comprising 61 preterm infants aged between 12 months (corrected age) and 65 months (chronological age). Language development was assessed using the raw scores of the ADL-2 Scale in receptive, expressive, and global modalities. Sociodemographic, peri-, and postnatal information was obtained through medical record analysis. Data were subjected to descriptive, association, and correlation analyses.

**Results:**

Descriptive analysis indicated lower language scores among male children, those from lower socioeconomic classes, those born via cesarean delivery, and those with a history of intracranial hemorrhage. No statistically significant associations or correlations were found.

**Conclusion:**

Although no statistically significant associations were observed, the descriptive findings suggest possible influences of sex, socioeconomic status, mode of delivery, and intracranial hemorrhage on the language performance of preterm infants. The results reinforce the importance of considering multiple factors in the language development of preterm children, suggesting longitudinal follow-up for timely interventions.

## INTRODUCTION

Prematurity is a major public health concern in Brazil, affecting approximately 11% of annual births and being the leading cause of infant mortality in the country^(1)^. The increased survival rate of premature infants, especially very low birth weight newborns, thanks to technical-scientific and technological advances, coupled with humanized neonatal care, has led to greater attention to peri- and postnatal occurrences, due to the possibility of short- and long-term sequelae, especially in preterm newborns (PTNB) with lower gestational age (GA)^(1)^. Furthermore, despite the higher survival rate of PTNBs, challenges persist due to clinical complications associated with prematurity, which increase the risk of neurodevelopmental disorders^(2)^. The literature discusses and emphasizes the importance of expanding pre- and postnatal assessments and care, not only during infancy, but also through longitudinal follow-up during the growth and development of preterm children^(2)^.

Disorders affecting language development are one of the possible effects of a history of prematurity, posing a risk for cognitive development as a whole^(3)^. A Brazilian scoping review mapped the evidence on risk and protective factors associated with early childhood development in children in Brazil and indicated that prematurity is a risk factor for neuropsychomotor development^(3)^, which includes language development in its receptive and expressive modalities.

In addition to impacts on language, another study presents results that indicate impairment in preterm children’s social interaction and quality of life^(4)^.

Language development can be approached overall or assessed individually through receptive and expressive modalities^(3)^, which may present distinct stages of development in the same premature child^(3)^. It has also been observed that the performance of each modality in language tests is influenced by the mother’s intrinsic and extrinsic factors^(5)^, the baby’s birth weight, the family’s socioeconomic classification, birth conditions, and GA and birth weight^(3)^, which are determinants frequently associated with the prognosis of neuropsychomotor development in premature children.

In the first years of life, which represent a crucial phase in human development, the prognosis of children born prematurely is influenced by the complex interaction of biological, social, economic, and environmental factors that act on the immature brain^(3)^. It is common to find studies that highlight the occurrence of neuropsychomotor development disorders, especially cognitive and language skills, associated with a history of prematurity and sociodemographic, peri- and postnatal factors^(6,7)^.

In the first minutes of life, the conditions at birth present determinants that can influence the PTNB’s development. In addition to GA and birth weight, factors such as mode of delivery, maternal age, APGAR (appearance, pulse, grimace, activity, respiration) scores, anthropometric measures, and the sex of the child add up to a range of risk or protective factors for their development^(7-11)^. The challenges of a favorable neurodevelopmental prognosis continue, since factors such as intracranial hemorrhage, length of stay, especially in the intensive care unit (NICU), and the socioeconomic context overlap with perinatal determinants^(7,12)^.

While studies frequently show an association between sociodemographic, peri- and postnatal factors with delayed language development in preterm children, studies such as that of Tseng and colleagues^(7)^, which are population-based, help to clarify the results of previous studies by questioning their data. The investigation of the said article was motivated by the authors' observation that many studies attempting to establish prematurity as the main risk factor for language delay did not obtain results consistent with related research^(7)^.

Thus, it is assumed that the language development of premature children is significantly influenced by sociodemographic, peri- and postnatal factors. Understanding the interaction of these factors and their impact on language development is a fundamental approach for intervention and prevention of unfavorable outcomes. Thus, this study aimed to verify the association between language development and sociodemographic, peri- and postnatal aspects of children with a history of prematurity attended in a follow-up clinic.

## METHODS

This is a preliminary observational, analytical, cross-sectional study with a non-probabilistic sample of 61 children with a history of prematurity, aged 12 months corrected age to 65 months chronological age. The study setting was the speech-language-hearing department of a follow-up clinic in a university hospital.

The study was approved by the University's Research Ethics Committee under approval number 3,615,440, and the parents or guardians of the participating children signed an informed consent form.

The inclusion criteria were children born prematurely and followed up by the clinic, aged 12 months corrected age to 65 months chronological age. Children diagnosed with autism spectrum disorder, sensorineural hearing loss, or cognitive, neurological, or psychiatric disorders were excluded.

Language data was collected with the Language Development Assessment 2 (ADL-2)^(13)^, which obtains raw receptive, expressive, and overall language scores. The psychometric data provided by the protocol classify language development quantitatively by the standard scores of the language modalities (receptive, expressive, and overall) only from 36 months of chronological age. To ensure homogeneous analysis of development in all individuals, regardless of age, the response variables were characterized by the raw receptive, expressive, and overall language scores. The raw receptive and expressive language scores are the number of tasks scored, minus the total number of tasks not scored, as instructed in the protocol. The raw overall language score is the sum of the raw scores of receptive and expressive language.

The children’s parents/guardians responded to the Brazilian Economic Classification Criteria (CCEB)^(14)^ to characterize socioeconomic aspects of their families. The economic classification is based on the family’s estimated purchasing power and the householder’s education level, ranging from classes A to E.

Data regarding peri- and postnatal factors were collected through the patient's physical or electronic medical record, namely: date of birth, child's age, chronological and corrected age (if applicable), sex, GA at birth, maternal age at the child's birth, mode of delivery, birth weight and length, head circumference, 1- and 5-minute APGAR scores, total length of stay, and occurrence of intracranial hemorrhage.

The dependent variables of the study were the receptive, expressive, and overall language scores. The independent variables were age (corrected or chronological), sex, GA, maternal age, mode of delivery, birth weight, birth length, head circumference, APGAR scores, length of stay, and occurrence of intracranial hemorrhage.

A descriptive analysis of the data was performed to meet the study objective, using the frequency distribution of categorical variables and analysis of measures of central tendency and dispersion of continuous variables. The Mann-Whitney and Kruskal-Wallis tests were used for association analyses. Non-parametric tests were used because the response variables did not present a normal distribution, confirmed by the Shapiro-Wilk and Kolmogorov-Smirnov tests, whose values were less than 0.05. Correlation analysis was also performed using Spearman's rank correlation coefficient, measuring the magnitude of the correlation based on the following parameters: weak = 0.0-0.4, moderate = 0.4-0.7, and strong = 0.7-1.0. Associations with a p-value ≤ 0.05 were considered significant. SPSS version 25.0 and Jamovi version 2.5 were used for data entry, processing, and analysis.

## RESULTS

The sample consisted of 61 children, 60.56% male and 39.34% female, with chronological ages ranging from 13 to 65 months (Mean 35.57 ± 13.86 SD) and corrected ages ranging from 12 to 23 months (Mean 17.93 ± 3.24 SD) (Table 1). Regarding socioeconomic strata, most of the sample was classified as C1 (42.62%), followed by C2 (29.50%), D-E (13.11%), and B2 (11.47%). The other classifications, A and B1, had only 1.63% of participants each.

**Table 1:** Characterization of the sample regarding peri- and postnatal factors

**Variables**	**N (%)**	**Mean**	**SD**	**Median**	**Minimum**	**Q_1_**	**Q_3_**	**Maximum**
Chronological age	61 (100.0)	35.57	13.86	32.00	13	24.50	50.00	65.00
Corrected age	14 (22.9)	17.93	3.24	18.00	12	16.50	20.00	23.00
Gestational age	61 (100.0)	31.43	2.35	32.00	25.00	30.00	33.00	35.00
Maternal age	61 (100.0)	30.98	7.63	31.00	14.00	24.00	37.50	45.00
Length at birth	61 (100.0)	38.62	5.61	39.00	14.00	35.00	42.00	56.00
Length of stay	61 (100.0)	45.34	34.85	35.00	7.00	23.00	58.50	188.00
Weight at birth	61 (100.0)	1471.00	434.29	1494.00	650.00	1197.50	1770.00	2740.00
Head circumference	59 (96.7)	28.02	2.92	28.00	22.00	26.00	29.00	37.00
1-minute APGAR	60 (98.3)	6.58	2.04	7.00	1.00	5.00	8.00	9.00
5-minute APGAR	60 (98.3)	8.47	1.28	9.00	4.00	8.00	9.00	10.00

**Caption:** N = number of individuals; SD = standard deviation; Q = quartile; % = percentage

**Figure 1:**
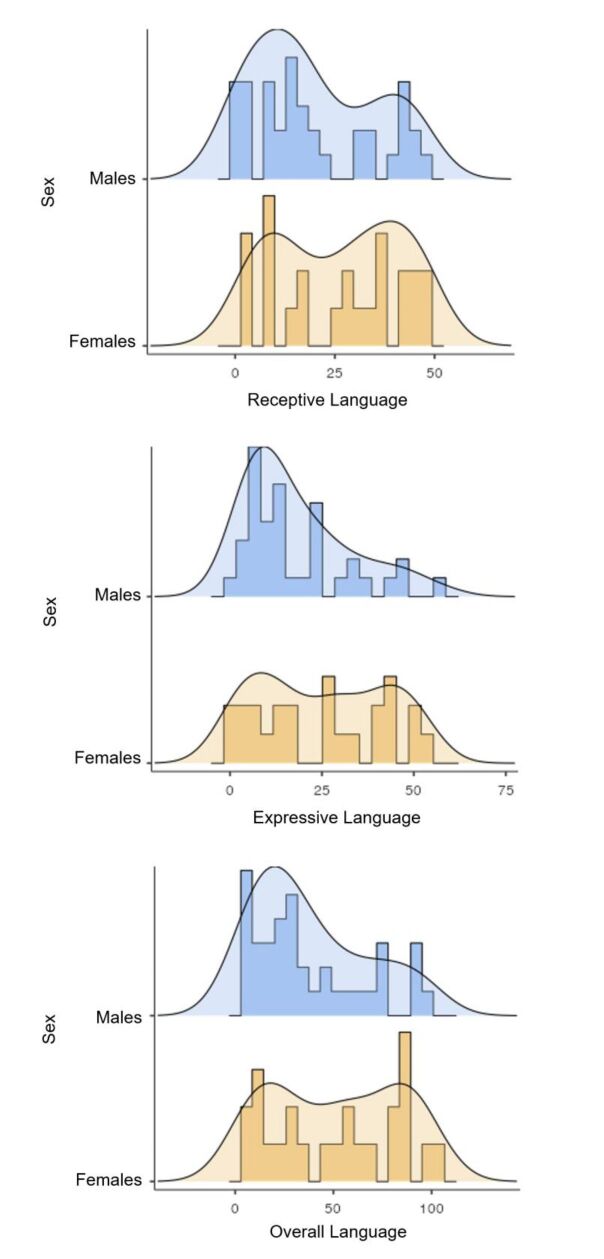
Density curves and violin plots of receptive, expressive, and overall language scores according to the sample's sex distribution

There was a prevalence of lower scores for each dependent variable among males, while higher language scores prevailed among females (Figure 1).

The characterization of peri- and postnatal factors (Table 1) showed that the mean GA of premature children was 31.43 weeks, with a minimum of 25 and a maximum of 35 weeks. The mothers were on average 30.98 years old (SD = 13.86) at their child’s birth, with an age range of 14 to 45 years. Regarding anthropometric measures at birth, the mean length was 38.62 cm, with minimum and maximum values ​​of 14 cm and 56 cm, respectively. Birth weight ranged from 650 grams to 2740 grams (Mean 1471.00 ± 434.29 SD). The mean head circumference was 28.02 cm (SD = 2.92). Regarding birth conditions, the children had an average of 6.58 on 1-minute and 8.47 on 5-minute APGAR scores, and the average length of stay was 45.34 days (SD = 34.85), with a minimum length of stay of 7 days and a maximum of 188 days.

Regarding the mode of delivery, 83.6% (n = 51) of the participants were born by cesarean section, while 16.3% (n = 10) were born vaginally. Intracranial hemorrhage occurred in 37.7% (n = 23) of the children, while most of them (62.3%) did not have this condition (Table 2).

**Table 2:** Characterization of the sample regarding peri- and postnatal factors

**Variables**	**N**	**%**
**Mode of delivery**		
Vaginal	10	16.3
Cesarean	51	83.6
Total	61	100.0
**Intracranial hemorrhage**		
Yes	23	37.7
No	38	62.3
Total	61	100.0

**Caption:** N = number of individuals; % = percentage

**Figure 2:**
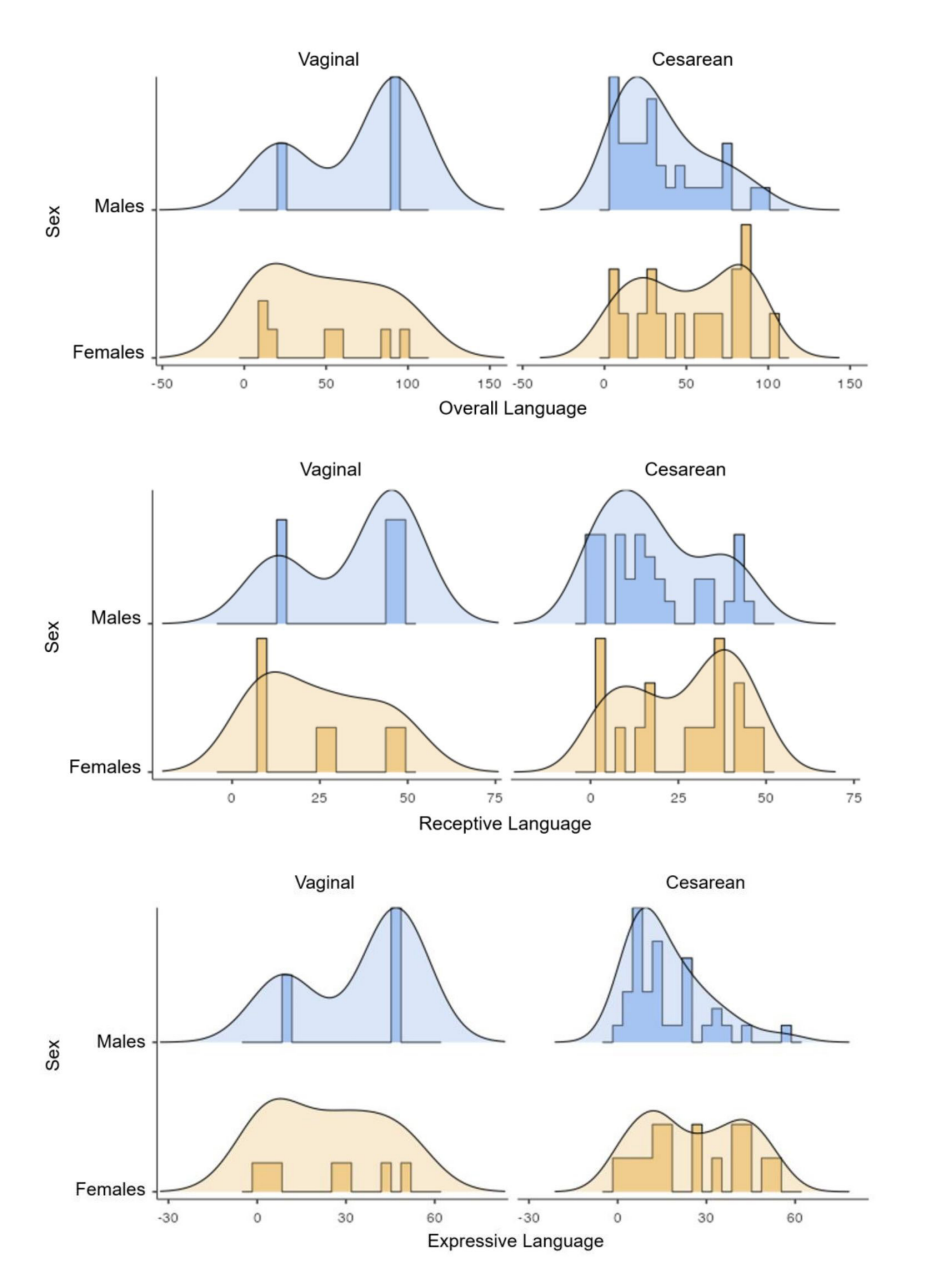
Density curves and histograms of the distribution of receptive, expressive, and overall language scores according to the mode of delivery

**Figure 3:**
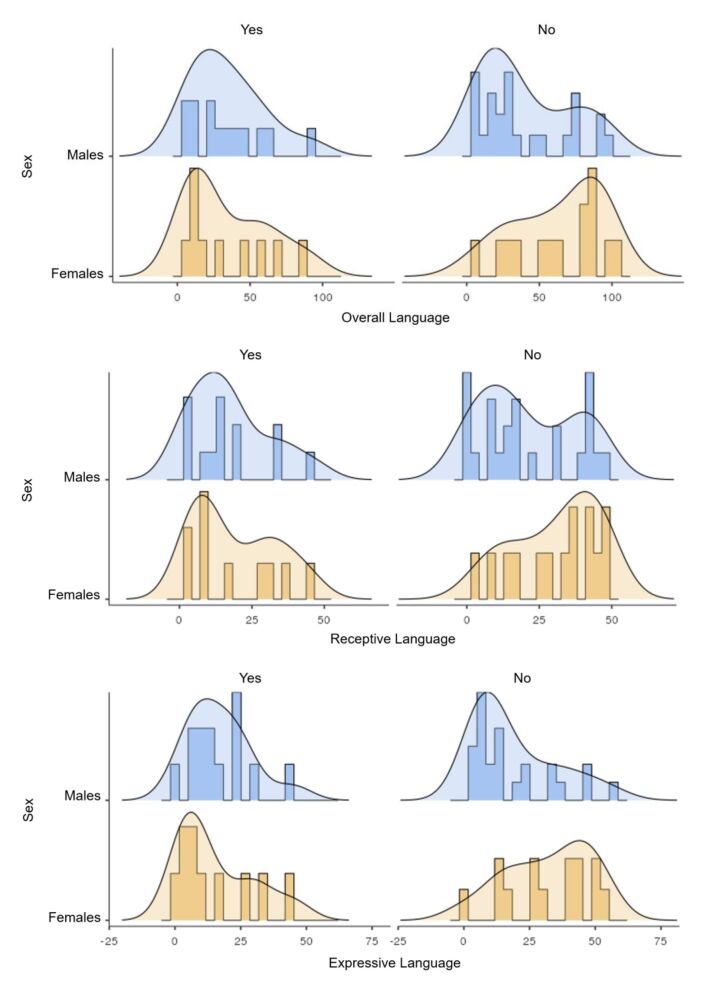
Density curves for receptive, expressive, and overall language scores according to the occurrence of intracranial hemorrhage

Among the children who underwent cesarean delivery, a higher density of high scores was observed in female premature children (Figure 2). In contrast, among children who experienced intracranial hemorrhage, the density of low scores was prevalent in premature infants regardless of sex, in all language modalities (Figure 3). As for those who did not experience this condition, there was a density of higher scores in females.

**Figure 4:**
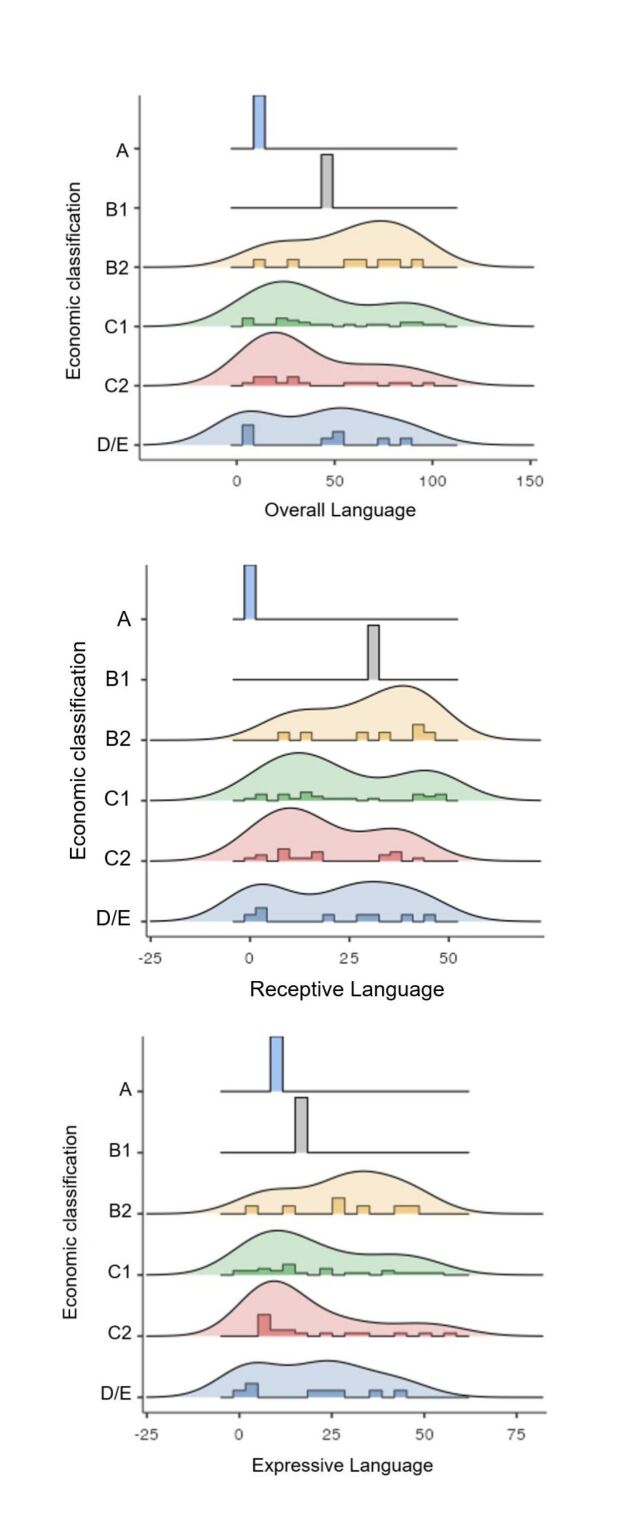
Density curves and histograms of the distribution of receptive, expressive, and overall language scores according to the socioeconomic classification of the sample

Regarding socioeconomic aspects, most families were classified as C1, representing 42.62% of the sample. The remaining participants in the sample were distributed as follows: one (1.63%) in category A, one (1.6%) in category B1, seven (11.4%) in category B2, 18 (29.5%) in category C2, and eight (13.1%) in category D-E. The evaluation of score distribution on the language performance test showed that premature children in lower economic classes scored lower (Figure 4).

The analysis of the general pattern of distribution of receptive, expressive, and overall language scores according to sex, economic classification, mode of delivery, and occurrence of intracranial hemorrhage revealed asymmetrical curves and, therefore, differences between the mean and median (Figures 1 to 4).

The language development assessment with ADL-2 revealed a mean receptive language score of 22.13, with scores ranging from 0 to 48. The minimum expressive language score was 0, and its maximum was 57 (mean of 20.84 ± 5.95 SD). The overall language score ranged from 3 to 101, with a mean of 43 (SD = 30.78).

No statistical significance was found in the association or correlation of sociodemographic factors and peri- and postnatal factors with language development variables (Tables 3, 4, and 5).

**Table 3:** Association between peri- and postnatal factors and language development

**Variables**	**Language Modality**									
	**n(%)**	**Receptive**			**Expressive**			**Overall**		
		**Mean±SD**	**Median**	**p-value**	**Mean±SD**	**Median**	**p-value**	**Mean±SD**	**Median**	**p-value**
**Mode of delivery**										
Vaginal	10 (16.3)	27.60±16.81	27.00	0.191	26.80±19.74	28.00	0.339	54.40±36.47	55.00	0.246
Cesarean	51 (83.6)	21.06±15.24	17.00		19.67±15.05	14.00		40.76±29.44	31.00	
**Intracranial hemorrhage**										
Yes	23 (37.7)	18.00±13.74	15.00	0.142	16.22±12.89	12.00	0.101	34.22±26.17	27.00	0.106
No	38 (62.2)	24.63±16.22	25.00		23.63±17.10	18.00		48.32±32.44	42.00	

Mann-Whitney U test

**Caption:** n = number of individuals; SD = standard deviation

**Table 4:** Association between sociodemographic factors and language development

**Variables**	**Language Modality**									
	**n(%)**	**Receptive**			**Expressive**			**Overall**		
		**Mean±SD**	**Median**	**p-value**	**Mean±SD**	**Median**	**p-value**	**Mean±SD**	**Median**	**p-value**
**Sex**										
Males	37 (60.6)	19.68±15.26	15.00	0.112	18.05±14.33	12.00	0.148	37.78±28.73	28.00	0.140
Females	24 (39.3)	25.92±15.55	27.50		25.13±17.54	27.00		51.04±32.69	54.50	
**CCEB**										
A/B	9 (14.7)	26.67±15.62	31.00	0.642	25.00±14.92	28.00	0.756	51.89±29.50	55.00	0.740
C1	26 (42.6)	23.04±16.51	16.50		20.85±16.47	14.50		43.88±32.30	32.00	
C2	18 (29.5)	18.94±13.91	15.50		19.17±16.58	11.50		38.11±29.71	26.50	
D-E	8 (13.1)	21.25±17.19	24.50		19.88±16.03	21.50		41.13±32.98	47.00	

Mann-Whitney U test

**Caption:** n = number of participants in the sample; SD = standard deviation

**Table 5:** Correlation between sociodemographic, peri- and postnatal factors and language development

**Variables**	**Receptive Language**	**Expressive Language**	**Overall Language**
Chronological age	0.888	0.839	0.890
Corrected age	0.236	0.535	0.545
Gestational age	0.056	0.035	0.035
Maternal age	0.020	0.041	0.031
Weight at birth	0.039	0.014	0.012
Length at birth	0.143	0.159	0.154
Head circumference	0.109	0.066	0.088
Length of stay	0.685	0.780	0.768
1-minute APGAR	0.226	0.292	0.250
5-minute APGAR	0.021	0.041	0.040

Spearman coefficient

## DISCUSSION

The language development of premature children is a crucial topic in child health, especially considering its possible medium- and long-term implications^(2,8)^. Factors such as male sex, low socioeconomic status, cesarean delivery, and intracranial hemorrhage, which are frequently cited as the main determinants of a restricted prognosis of language development^(3)^, proved to be influential for lower language performance in the sample of this study.

Male preterm children were at higher risk of short- and long-term neurodevelopmental, cognitive, and motor impairment and had a greater tendency to abnormal white matter development^(15)^. The sample distribution showed that the receptive, expressive, and overall language scores revealed a higher density of lower scores in males. This corroborates previous studies that indicate a greater number of boys with impaired language development^(7,9)^. Moreover, females had a symmetrical distribution of such scores, which corroborates the results of the said studies^(7,9)^.

A study investigating the risk factors of prematurity and its relationship with language development indicated the importance of assessment in the first 24 months with corrected age^(7)^. In its study sample, corrected age was considered up to 23 months and 29 days, representing almost a quarter of participants. Chronological age, for analysis, was considered from 24 months of age. Regardless of the scenario, this study does not corroborate previous results, either in studies with children between 0 and 24 months of corrected age^(7,16)^ or in studies with chronological age equal to or greater than 24 months^(9,16,17)^, which showed that the age of the premature infant was statistically significantly associated with delay in language development.

The clinical profile of prematurity found in the sample is frequently associated with language delay, as indicated in the literature^(7)^. Considering the average GA and birth weight obtained, according to data from the World Health Organization, the sample profile can be classified as severe prematurity and very low birth weight. Extremely premature (GA < 28 weeks) and very low birth weight newborns have been associated with moderate and severe language delays^(7)^. However, no statistically significant difference was found between the GA of extremely and severely PTNBs and the effects on language development^(18)^. This suggests similar risks of abnormal language development when considering birth before 32 weeks of GA.

The study found no statistically significant association between weight, GA, and language development scores, which contrasts with the findings of other studies^(7,18)^. This research assessed language variables based on instrument scores without categorizing the severity of language impairment, potentially explaining why it did not confirm previous results. The variation among premature individuals with different weights and GA at birth indicates that these groups do not share uniform development. This presents a challenge in establishing a clear cause-and-effect relationship between prematurity, birth weight, GA, and language development. It also underscores the importance of exploring protective mechanisms that might lessen the negative impacts of risks on the development of premature infants^(17)^.

Recent studies diverge regarding the influence of head circumference and length measures on the impact on neurodevelopment^(19,20,21)^. While one study observed that head circumference had an unfavorable outcome for neurocognitive development at 6 months of age^(22)^, another found no relationship between these variables^(20)^, as did a recent study, which found no relationship between length and neurodevelopment^(21)^. The term length is indirectly related to birth weight and GA, and its use is not common in previous studies^(21)^.

Socioeconomic class is a variable of great interest to researchers regarding risk factors for the development not only of language but of the entire neuropsychomotor apparatus in preterm children. However, despite the frequent statistically significant association between socioeconomic class and language development, there is considerable variability in different studies^(17,18)^. A literature review with meta-analysis showed that five of the seventeen studies analyzed did not present a statistically significant association between language development and socioeconomic class^(17)^. More than 40% of the families of the children evaluated were classified in category C1, which infers an average gross income of 2.2 minimum wages per family^(14)^. This percentage is above the general estimate for the municipality where the follow-up clinic is located, which is 23.4%, according to the validation and dissemination document of this instrument^(14)^. In this sample, more than two-thirds of families were classified in classes C1 and C2, which obtained lower receptive, expressive, and overall language scores, corroborating studies that say that unfavorable socioeconomic context is related to lower language performance^(7,12)^. The possibility of different outcomes in language development when considering the socioeconomic factor was previously highlighted^(17)^. Recent studies^(9,15)^ present similar results, suggesting that female sex, combined with better socioeconomic conditions, results in better performance in language tests. In the context of this study, no statistically significant association was obtained between economic classification and any dependent variable. However, the results strengthen other studies that show a strong influence of socioeconomic context on low language performance.

Maternal age at birth and cesarean delivery are factors that influence unfavorable outcomes for premature children^(22)^. A population-based study^(2)^ found that maternal age above 35 years was statistically significantly associated with delayed language development in PTNBs. Martins et al.^(3)^ mentioned in a scope review that premature children of adolescent mothers were at higher risk for cognitive development, which includes language development. The analysis suggests that the extremes of a woman's fertile age show greater associations with delayed language development. In our sample, the average maternal age at birth was between the extremes, which may justify the absence of a statistically significant association with language development.

The study findings show a prevalence of cesarean deliveries, corroborating a study in which this method is prevalent among the deliveries of children with a history of prematurity^(18)^. Women who experience complications during pregnancy are at higher risk of emergency cesarean sections and the premature birth of their babies^(23)^, since cesarean delivery, when well indicated, helps to reduce perinatal risks and increase the survival of newborns^(24)^. The literature has reported an association between vaginal delivery and better performance on language tests in premature children^(3)^. The data analysis in this study found no statistically significant association between these two variables. However, cesarean delivery had a higher density of lower scores in males in all language modalities studied, indicating that vaginal birth and female sex predispose to better performance on language development tests.

The mean 1- and 5-minute APGAR scores in our sample suggest a gradual adaptation to the extrauterine environment^(25)^. A recent study observed normal 1-minute APGAR scores (i.e., equal to or greater than 7) in premature children born with GA between 28 and 37 weeks, not establishing a significant relationship with language development^(8)^, as in the present study. However, the literature also shows that low 1-minute APGAR scores were associated with language disorders^(23)^, while another study pointed to the statistical significance of lower 1- and 5-minute APGAR scores with language disorders^(26)^. The divergence between studies indicates the need for further investigation into the impact of APGAR scores on language development.

The large variability in the sample's length of stay demonstrates important differences between participants. Prolonged NICU stay of premature infants poses a risk for neurodevelopment^(8,25)^, as it restricts important stimuli for the beginning of neuropsychomotor maturation, which is significantly related to unfavorable outcomes for cognition, language, and motor skills before 12 months of age^(25)^.

The presence of intracranial hemorrhage in just over a third of the sample differs from a study that revealed that this condition is not so prevalent in premature infants^(8)^. However, its occurrence in moderate and severe degrees is strongly related to language delay^(7)^. The present study found no statistically significant association between intracranial hemorrhage and language development, but females who had this condition had lower scores, and those who did not have it had higher scores. In males, language scores were lower regardless of having this condition. These findings confirm that intracranial hemorrhage is a predisposing factor for delayed language development, but other factors may have a greater influence on this clinical outcome.

Previous studies^(27)^ have pointed out the discrepancy in preterm children’s development of receptive and expressive language. Although considering scores from other language assessment instruments, these studies indicated lower performance of language skills in the expressive modality than in the receptive modality among preterm children. Notably, one of these studies identified a statistically significant difference between the subtests of the two language modalities^(27)^. The distribution of scores of both modalities in the sample of the present study showed a lower mean in expressive than in receptive language. This finding corroborates the aforementioned studies and is consistent with language development stages, which predict that comprehension precedes production. Thus, the development of receptive skills is a basis for the development of expressive language skills^(27)^.

None of the study's dependent variables was statistically significantly associated with any sociodemographic variables or peri- and postnatal factors, although some independent variables influenced language development variables. The sample size and design stand out as limitations of the study, making it impossible to generalize the results to other contexts beyond the service that was the study setting. The sample size also prevented the grouping of participating children into similar age ranges due to the heterogeneous ages in the sample. Hence, further research should use this language assessment tool and focus on standardized metrics. It is essential to expand the sample, seeking better distribution among age ranges to homogenize the analysis of the results.

On the other hand, this study included the analysis of several variables that have been previously identified in the literature as influential in language development. Furthermore, the sample consisted of premature children followed up in an outpatient service, which allowed for a detailed survey of the risk factors analyzed. Another important point is that the socioeconomic context of the families was considered, which is being increasingly investigated by related studies due to its significant influence on the spectrum of neuropsychomotor development.

Timely assessment and longitudinal monitoring of language development aim to improve the quality of life of preterm children. Some study results do not corroborate the findings of other studies^(5-11)^, which indicated that language development is significantly related to sociodemographic, peri-, and postnatal conditions. However, periodic, standardized, rigorous assessments are justifiable and necessary to identify possible problems in child development from the beginning of development.

## CONCLUSION

Despite the absence of statistically significant associations between sociodemographic, peri-, and postnatal factors and the language development of premature children followed up in an outpatient clinic, the findings of this preliminary study point to important trends. Lower receptive, expressive, and overall language scores were more highly concentrated among male children and those belonging to lower socioeconomic strata. These results, while not conclusive, reinforce the relevance of a multidimensional approach in the assessment of the language development of premature children, highlighting the need for future studies with larger samples and analytical designs that can deepen the understanding of these complex relationships. Finally, it is important to emphasize the importance of longitudinal follow-up, with periodic assessments of language development to identify possible impairments early and propose appropriate interventions to ensure the best support for development and promote quality of life for premature children in the long term.

## References

[1] Albertoni M, Rosa VM, Iser BPM (2023). Prevalência e tendência temporal da prematuridade no Brasil antes e durante a pandemia de covid-19: análise da série histórica 2011-2021. SciELO Preprints.

[2] Maia AAA, Pinto APO, Viana JN, Sousa GA, Mourão GG (2022). Fatores de risco da prematuridade: uma revisão narrativa. REAS.

[3] Martins IM, Perazzo MF, Corrêa-Faria P, Santos IG, Mateus AC, Fernandez AM (2025). Fatores de risco e proteção no desenvolvimento infantil na primeira infância: uma revisão de escopo. Rev Bras Saúde Mater Infant.

[4] Vieira MEB, Linhares MBM (2011). Developmental outcomes and quality of life in children born preterm at preschool- and school-age. J Pediatr (Rio J).

[5] Cunha AMT, Fernandes Y, Pinto CF, Santos JS, Rodrigues OMPR (2022). Desenvolvimento de prematuros: efeito da saúde emocional materna e de uma intervenção psicoeducativa. Saúde e Desenvolvimento Humano.

[6] Dias LBT, Rubini EDC (2022). Características neuropsicológicas do desenvolvimento de bebês prematuros e a termo: uma revisão da literatura. Estud Pesqui Psicol.

[7] Tseng WL, Chen CH, Chang JH, Peng CC, Jim WT, Lin CY (2023). Fatores de risco de atraso de linguagem aos dois anos de idade corrigida entre bebês prematuros com muito baixo peso ao nascer: um estudo de base populacional. Children.

[8] Teixeira MA, Britto DBO, Escarce AG, Paula DD, Lemos SMA (2022). Perfil de prematuros em atendimento fonoaudiológico em um ambulatório de follow up. Audiol Commun Res.

[9] Lingappan K, Alur P, Eichenwald E (2023). The need to address sex as a biological variable in neonatal clinical studies. J Pediatr.

[10] Oliveira SR, Machado ACCP, Magalhães LC, Miranda DM, Paula JJ, Bouzada MCF (2023). Cognitive assessment in preterms by Bayley-III: development in the first year and associated factors. Rev Paul Pediatr.

[11] Leitão FNC, Torres JGS, Dias ERSD, Torres JST, Lopes AGPL, Ferreira CRT (2023). Escala de Apgar em recém-nascidos prematuros: revisão sistemática. Revista Multidisciplinar em Saúde..

[12] El-Din EMS, Elabd MA, Nassar MS, Metwally AM, Abdellatif GA, Rabah TM (2019). The interaction of social, physical and nutritive factors in triggering early developmental language delay in a sample of Egyptian children. Open Access Maced J Med Sci.

[13] Menezes ML (2021). ADL: Avaliação do desenvolvimento da linguagem..

[14] ABEP: Associação Brasileira de Empresas de Pesquisa (2022). Critério de Classificação Econômica Brasil – CCEB 2022.

[15] Macedo I, Pereira-da-Silva L, Brito L, Cardoso M (2019). Male sex is an independent risk factor for poor neurodevelopmental outcome at 20 months’ corrected age, in human milk-fed very preterm infants: a cohort study. Einstein (Sao Paulo).

[16] Gouveia AS, Oliveira MMF, Goulart AL, Azevedo MF, Perissinoto J (2020). Desenvolvimento de linguagem e das habilidades auditivas em prematuros adequados e pequenos para a idade gestacional: idade cronológica entre 18 e 36 meses. CoDAS.

[17] Van Noort-van der Spek IL, Franken MC, Weisglas-Kuperus N (2012). Language functions in preterm-born children: a systematic review and meta-analysis. Pediatrics.

[18] Kara ÖK, Günel MK, Açıkel C, Yiğit Ş, Arslan M (2015). Is there any difference between high-risk infants with different birth weight and gestational age in neurodevelopmental characters?. Turk Pediatri Ars.

[19] Oliveira SR, Machado ACCP, Magalhães LC, Miranda DM, Paula JJ, Bouzada MCF (2023). Cognitive assessment in preterms by Bayley-III: development in the first year and associated factors. Rev Paul Pediatr.

[20] Hass JV, Panceri C, Procianoy RS, Silveira RC, Valentini NC (2023). Risk factors for cognitive, motor and language development of preterm children in the first year of life. Rev Paul Pediatr.

[21] Panceri C, Valentini NC, Silveira RC, Smith BA, Procianoy RS (2020). Neonatal adverse outcomes, neonatal birth risks, and socioeconomic status: combined influence on preterm infants’ cognitive, language, and motor development in Brazil. J Child Neurol.

[22] Gomes TGACB, Queiroz MN, Costa ABMP, Moreira ACG (2021). Desfechos perinatais relacionados à idade materna e comorbidades gestacionais nos nascimentos prematuros. Comunicação em Ciências da Saúde.

[23] Souza DML, Silva Maia LC, Zêgo ZDF, Jaeger GP, Maciel WS (2019). Prevalência de prematuridade e fatores associados no estado do Rio Grande do Sul. Braz J Hea Rev..

[24] Toneli LS, da Silva MB, Pinto AAM, Queiroz FC, de Queiroz LMP (2024). Influence of biopsychosocial factors on development of premature and full-term babies. RSD.

[25] Silva MF, Rozeira CHB, Oliveira RS, Matos AAL, Carneiro PEC, Costa JC (2024). Impacto da prematuridade no desenvolvimento neuropsicomotor infantil. Brazilian Journal of Implantology and Health Sciences..

[26] Nascimento GB, Kessler TM, Souza APR, Costa I, Moraes AB (2020). Indicadores de risco para a deficiência auditiva e aquisição da linguagem e sua relação com variáveis socioeconômicas, demográficas e obstétricas em bebês pré-termo e a termo. CoDAS.

[27] Cusson RM (2003). Factors influencing language development in preterm infants. J Obstet Gynecol Neonatal Nurs.

