# Supplementary material for: Language development of premature children followed up in an outpatient clinic: a preliminary study
Source: Codas. 2026 Apr 10;38(2):e20240284. doi: 10.1590/2317-1782/e20240284en (PMC13075844; doi:10.1590/2317-1782/e20240284en)
Supplement: Translation

# Translation: Desenvolvimento linguístico de prematuros acompanhados em um ambulatório de follow-up: estudo preliminar

**DOI:** 10.1590/2317-1782/e20240284pt

**Authors:** Jonathan Gonçalves Rocha, Maisa Alves Teixeira, Andrezza Gonzalez Escarce, Thamara Suzi dos Santos, Denise Brandão de Oliveira e Britto, Stela Maris Aguiar Lemos

**Affiliations:** 1 Departamento de Fonoaudiologia, Faculdade de Medicina, Universidade Federal de Minas Gerais – UFMG - Belo Horizonte (MG), Brasil.; 2 Programa de Pós-graduação em Ciências Fonoaudiológicas, Faculdade de Medicina, Universidade Federal de Minas Gerais – UFMG - Belo Horizonte (MG), Brasil.

**Keywords:** Nascimento Prematuro, Linguagem Infantil, Desenvolvimento da Linguagem, Criança, Diagnóstico

## Abstract

**Objetivo:**

Verificar a associação entre o desenvolvimento de linguagem e aspectos sociodemográficos, peri e pós-natais de crianças com histórico de prematuridade acompanhadas em um ambulatório de follow-up.

**Método:**

Estudo observacional, analítico, transversal e preliminar composto 61 prematuros entre 12 meses (idade corrigida) e 65 (idade cronológica). O desenvolvimento linguístico foi avaliado por meio dos escores brutos da Escala ADL-2, nas modalidades receptiva, expressiva e global. As informações sociodemográficas, peri e pós-natais foram obtidas por meio de análise de prontuários. Os dados foram submetidos a análises descritiva, de associação e de correlação.

**Resultados:**

A análise descritiva indicou menores escores de linguagem entre as crianças do sexo masculino, de classes socioeconômicas mais baixas, nascidas por parto cesáreo e com histórico de hemorragia intracraniana. Não foram encontradas associações ou correlações estatisticamente significativas.

**Conclusão:**

Embora não tenham sido observadas associações com significância estatísticas, os achados descritivos apontam possíveis influências do sexo, condição socioeconômica, via de parto e hemorragia intracraniana no desempenho linguístico de prematuros. Os resultados reforçam a importância de considerar múltiplos fatores no desenvolvimento linguístico de prematuros, sugerindo acompanhamento longitudinal para intervenções em tempo oportuno.

## INTRODUÇÃO

No Brasil, a prematuridade representa grande preocupação para a saúde pública visto que afeta cerca de 11% dos nascimentos anuais, sendo a principal causa de mortalidade infantil no país^(1)^. O aumento da taxa de sobrevivência de prematuros, especialmente recém nascidos de muito baixo peso, graças ao avanço técnico-científico e tecnológico, aliado à assistência neonatal humanizada, tem proporcionado maior atenção às ocorrências nos períodos peri e pós-natais, devido à possibilidade de sequelas a curto e longo prazo, especialmente em recém nascidos pré-termo (RNPT) com menor idade gestacional (IG)^(1)^. Além disso, apesar da maior taxa de sobrevivência dos nascidos pré-termo, os desafios persistem devido às complicações clínicas associadas à prematuridade, que aumentam o risco para transtornos do neurodesenvolvimento^(2)^. Na literatura, é debatida e enfatizada a importância de ampliar as avaliações e os cuidados pré e pós-natais, não apenas durante a infância, mas por meio de acompanhamento longitudinal durante o crescimento e desenvolvimento dos pré-termos^(2)^ **.**

Transtornos que afetam o desenvolvimento da linguagem são um dos possíveis efeitos decorrentes do histórico de prematuridade, além de representar risco para o desenvolvimento cognitivo como um todo^(3)^. Uma revisão de escopo brasileira mapeou as evidências sobre os fatores de risco e proteção associados ao desenvolvimento na primeira infância de crianças no Brasil e indicou que a prematuridade é um fator de risco para o desenvolvimento neuropsicomotor^(3)^, o que inclui o desenvolvimento linguístico em suas modalidades receptiva e expressiva.

Além dos impactos linguísticos, outro estudo apresenta resultados que indicam prejuízo no convívio social e na qualidade de vida de prematuros^(4)^.

O desenvolvimento linguístico, além de ser compreendido de uma forma global, também pode ser avaliado por meio das modalidades receptiva e expressiva, individualmente^(3)^, que podem apresentar etapas distintas do desenvolvimento no mesmo indivíduo prematuro^(3)^. Além disso, também foi observado que o desempenho de cada modalidade nos testes linguísticos sofre influência de fatores intrínsecos e extrínsecos da mãe^(5)^, mas também de peso ao nascimento, classificação socioeconômica da família, condições de nascimento, idade gestacional e peso ao nascer^(3)^, que são determinantes frequentemente associados ao prognóstico do desenvolvimento neuropsicomotor de prematuros.

Nos primeiros anos de vida, que representam uma fase crucial no desenvolvimento humano, o prognóstico das crianças nascidas prematuras é influenciado pela complexa interação dos fatores biológicos, sociais, econômicos e ambientais que atuarão no cérebro imaturo^(3)^. É comum encontrar estudos que destacam a ocorrência de transtornos do desenvolvimento neuropsicomotor, sobretudo de habilidades cognitivas e linguísticas associadas ao histórico de prematuridade, bem como fatores sociodemográficos, peri e pós-natais^(6,7)^.

Nos primeiros minutos de vida, as condições ao nascimento apresentam determinantes que podem vir a influenciar o desenvolvimento deste RNPT. Além da IG e o peso ao nascer, fatores como via de parto, idade materna, escores APGAR, medidas antropométricas, bem como o sexo da criança, somam uma gama de fatores de risco ou protetivos para seu desenvolvimento^(7-11)^. Os desafios de um prognóstico favorável ao neurodesenvolvimento ainda se seguem, visto que fatores como ocorrência de hemorragia intracraniana; tempo de internação, sobretudo na Unidade de Terapia Intensiva (UTIN), e o contexto socioeconômico se sobrepõem aos determinantes peri-natais^(7,12)^.

Ao passo que, frequentemente, estudos apresentam associação entre os fatores sociodemográficos, peri e pós-natais com o atraso no desenvolvimento linguístico de prematuros, estudos como o de Tseng e colaboradores^(7)^, que são de base populacional, contribuem para esclarecer os resultados de estudos anteriores, ao questionar os dados provenientes destes. No artigo referido a investigação foi motivada pela observação dos autores de que muitos estudos que tentaram estabelecer a prematuridade como o principal fator de risco para o atraso da linguagem não obtiveram resultados consistentes com pesquisas correlatas^(7)^.

Deste modo, pressupõe-se que o desenvolvimento linguístico de prematuros apresenta influência significativa dos fatores sociodemográficos, peri e pós-natais. Compreender a interação desses fatores e de seu impacto no desenvolvimento linguístico é uma abordagem fundamental para intervenção e prevenção de desfechos desfavoráveis. Portanto, o objetivo deste estudo foi verificar a associação entre o desenvolvimento de linguagem e aspectos sociodemográficos, peri e pós-natais de crianças com histórico de prematuridade acompanhadas em um ambulatório de *follow-up*.

## MÉTODO

Trata-se de um estudo observacional, analítico e transversal preliminar, com amostra não probabilística, composta por 61 crianças com histórico de prematuridade na faixa etária entre 12 meses de idade corrigida a 65 meses de idade cronológica. O cenário do estudo foi o setor de Fonoaudiologia de um ambulatório de *follow-up*, situado em um hospital universitário.

O estudo teve aprovação do Comitê de Ética em Pesquisa da Universidade sob o parecer de número 3.615.440 e os pais ou responsáveis das crianças participantes assinaram o Termo de Consentimento Livre e Esclarecido (TCLE).

Os critérios de inclusão foram crianças nascidas prematuras e acompanhadas pelo ambulatório de *follow-up* com faixa etária entre 12 meses de idade corrigida a 65 meses de cronológica. Foram excluídas crianças diagnosticadas com Transtorno do Espectro Autista (TEA), com perda auditiva sensório-neural, com alterações cognitivas, neurológicas ou psiquiátricas.

Para coleta dos dados linguísticos foi aplicado o protocolo Avaliação do Desenvolvimento da Linguagem 2 (ADL-2)^(13)^ que tem como objetivo a obtenção dos escores brutos das modalidades receptiva e expressiva da linguagem, bem como o escore bruto da linguagem global. Os dados psicométricos fornecidos pelo protocolo classificam o desenvolvimento da linguagem de forma quantitativa pelos escores padrões das modalidades da linguagem (receptiva, expressiva e global), somente a partir dos 36 meses de idade cronológica; e, para manter a homogeneidade da análise do desenvolvimento em todos os indivíduos, independentemente da idade, considerou-se que as variáveis respostas fossem caracterizadas pelo escore bruto das linguagens receptiva, expressiva e global. Os escores brutos das modalidades receptiva e expressiva da linguagem referem-se à quantidade de tarefas pontuadas, menos o total de tarefas não pontuadas, como instruído no protocolo. O escore bruto da linguagem global refere-se à soma dos escores brutos das modalidades receptiva e expressiva da linguagem.

Para caracterizar o aspecto socioeconômico das famílias, os responsáveis pelas crianças responderam ao Critério de Classificação Econômica Brasil - CCEB^(14)^. A classificação econômica é pautada na estimativa do poder aquisitivo e do grau de instrução do chefe da família, variando entre as classes A e E.

Os dados referentes aos fatores peri e pós-natais foram coletados por meio do prontuário físico ou eletrônico do paciente, a saber: data de nascimento; idade da criança, cronológica e corrigida (caso se aplicasse); sexo; idade gestacional ao nascimento; idade materna ao nascimento da criança; via de parto; peso ao nascer; estatura; perímetro cefálico; APGAR (primeiro e quinto minuto); tempo total de internação e ocorrência de hemorragia intracraniana.

As variáveis dependentes do estudo foram os escores de linguagem receptiva, linguagem expressiva e linguagem global. As variáveis independentes foram idade (corrigida ou cronológica), sexo, idade gestacional, idade materna, via de parto, peso ao nascer, estatura, perímetro cefálico, APGAR, tempo de internação e ocorrência de hemorragia intracraniana.

Para atender ao objetivo do estudo foi realizada a análise descritiva dos dados, por meio da distribuição de frequência das variáveis categóricas e análise das medidas de tendência central e de dispersão das variáveis contínuas. Para as análises de associação foram utilizados os testes Mann-Whitney e Kruskal-Wallis. A escolha dos testes não paramétricos deve-se ao fato das variáveis resposta não apresentarem uma distribuição normal, confirmada por meio dos testes dos testes Shapiro Wilk e Kolmogorov-Smirnov, cujos valores encontrados foram menores que 0,05. Também foi realizada análise de correlação, por meio do coeficiente de correlação de Spearman, no qual a magnitude da correlação foi medida seguindo o seguinte parâmetro: fraca = 0,0-0,4; moderada = 0,4-0,7 e forte = 0,7-1,0. Foram consideradas como associações significantes as que apresentaram valor de p≤0,05. Para entrada, processamento e análise dos dados foram utilizados os softwares SPSS, versão 25.0, e Jamovi, versão 2.5.

## RESULTADOS

A amostra foi composta por 61 crianças, sendo 60,56% do sexo masculino e 39,34% do sexo feminino com variação da idade cronológica de 13 a 65 meses (Média 35,57 ± 13,86 DP) e de idade corrigida de 12 e 23 meses (Média 17,93 ± 3,24 DP) (tabela 1). Quanto aos estratos socioeconômicos, a maior parte da amostra foi classificada como C1(42,62%), seguido de C2 (29,50%), D-E (13, 11%) e B2(11,47%). As demais classificações, A e B1, contaram com apenas 1,63% de participantes em cada estrato socioeconômico.

**Tabela 1:** Caracterização da amostra quanto fatores peri e pós-natais

**Variáveis**	**N (%)**	**Média**	**D.P.**	**Mediana**	**Mínimo**	**1º Q**	**3º Q**	**Máximo**
Idade cronológica	61 (100,0)	35,57	13,86	32,00	13	24,50	50,00	65,00
Idade corrigida	14 (22,9)	17,93	3,24	18,00	12	16,50	20,00	23,00
Idade gestacional	61 (100,0)	31,43	2,35	32,00	25,00	30,00	33,00	35,00
Idade materna	61 (100,0)	30,98	7,63	31,00	14,00	24,00	37,50	45,00
Estatura	61 (100,0)	38,62	5,61	39,00	14,00	35,00	42,00	56,00
Tempo de Internação	61 (100,0)	45,34	34,85	35,00	7,00	23,00	58,50	188,00
Peso ao nascer	61 (100,0)	1471,00	434,29	1494,00	650,00	1197,50	1770,00	2740,00
Perímetro cefálico	59 (96,7)	28,02	2,92	28,00	22,00	26,00	29,00	37,00
APGAR 1’	60 (98,3)	6,58	2,04	7,00	1,00	5,00	8,00	9,00
APGAR 5’	60 (98,3)	8,47	1,28	9,00	4,00	8,00	9,00	10,00

**Legenda:** N = número de indivíduos; D.P. = desvio padrão; Q = quartil; % = percentual

No sexo masculino, observou-se que, para cada variável dependente, houve prevalência de escores mais baixos, enquanto no sexo feminino, prevaleceu escores de linguagem maiores (Figura 1).

**Figura 1:** Curvas de densidade e *vionlin plots* dos escores das modalidades receptiva, expressiva e global da linguagem segundo a distribuição por sexo da amostra

A caracterização dos fatores peri e pós-natais (Tabela 1), mostrou que a média da idade gestacional dos prematuros foi de 31,43 semanas, com mínimo de 25 semanas e máximo de 35. As mães tinham, ao nascimento do filho, em média 30,98 anos de idade (DP=13,86), com variação de idade de 14 a 45 anos. Quanto às medidas antropométricas ao nascimento, a média da estatura foi de 38,62 cm, com valores mínimos e máximos de 14 cm e 56 cm, respectivamente. O peso ao nascer variou de 650 gramas a 2740 gramas (Média 1471,00 ± 434,29 DP). A média do perímetro cefálico foi de 28,02 (DP = 2,92). Em relação às condições de nascimento, na Escala de *Appearance*, *Pulse, Grimace, Activity, Respiration* (APGAR), as crianças apresentaram média de 6,58 no primeiro minuto e 8,47 no quinto minuto, e o tempo médio de internação foi de 45,34 dias (DP = 34,85), com o tempo mínimo de internação de 7 dias e o máximo 188 dias.

No que se refere à via de parto 83,6% (n = 51) dos participantes nasceram por cesárea, enquanto 16,3% (n = 10), nasceram por via de parto vaginal. A ocorrência de hemorragia intracraniana esteve presente em 37,7% (n = 23) das crianças, enquanto a maior parte deles (62,3%) não tiveram essa condição (Tabela 2).

**Tabela 2:** Caracterização da amostra quanto a fatores peri e pós-natais

**Variáveis**	**N**	**%**
**Via de parto**		
Vaginal	10	16,3
Cesárea	51	83,6
Total	61	100,0
**Hemorragia intracraniana**		
Sim	23	37,7
Não	38	62,3
Total	61	100,0

**Legenda:** N = número de indivíduos; % = percentual

Das crianças que apresentaram via de parto cesárea, foi observada maior densidade de escores elevados em prematuros do sexo feminino (Figura 2). Em contrapartida, nas crianças que apresentaram ocorrência de hemorragia intracraniana, a densidade de escores baixos foi prevalente nos prematuros, independente do sexo, em todas as modalidades linguísticas (Figura 3). Quanto às crianças que não apresentaram ocorrência desta condição, houve densidade de maiores escores no sexo feminino.

**Figura 2:** Curvas de densidade e histogramas da distribuição dos escores das modalidades receptiva, expressiva e global da linguagem segundo à via de parto

**Figura 3:** Curva de densidade de escores das modalidades receptiva, expressiva e global da linguagem segundo a ocorrência de hemorragia intracraniana

Quanto ao aspecto socioeconômico, verificou-se que a maior parte das famílias foi classificada como C1, representada por 42,62% da amostra; os demais participantes da amostra foram distribuídos da seguinte forma: 1 (1,63%) na categoria A, 1 (1,6%) na categoria B1, 7 (11,4%) na categoria B2, 18 (29,5%) na categoria C2 e 8 (13,1%) na categoria D-E. Ao avaliar a distribuição dos escores do teste de desempenho linguístico, foi notado que nas classes econômicas mais baixas os prematuros pontuaram menos (Figura 4).

**Figura 4:** Curvas de densidade e histogramas da distribuição dos escores das modalidades receptiva, expressiva e global da linguagem segundo classificação socioeconômica da amostra

A análise do padrão geral da distribuição dos escores de linguagem receptiva, expressiva e global segundo sexo, classificação econômica, via de parto e ocorrência de hemorragia intracraniana revelou curvas assimétricas e, portanto, com diferenças entre média e mediana (Figuras 1 a 4).

A avaliação do desenvolvimento da linguagem realizada pelo protocolo ADL-2 revelou que, para a modalidade receptiva da linguagem a média de escore foi 22,13 com variação de escore entre 0 e 48. Na modalidade expressiva, o escore mínimo obtido foi 0 e o máximo 57 (média 20,84 ± 5,95 DP). No escore global da linguagem, os escores variaram entre 3 e 101, e a média de escores obtidos foi de 43 (DP = 30,78).

Não foi observada significância estatística na associação ou na correlação dos fatores sociodemográficos e fatores peri e pós-natais com as variáveis de desenvolvimento linguístico (Tabelas 3, 4 e 5).

**Tabela 3:** Associação entre fatores peri e pós-natais e desenvolvimento linguístico

**Variáveis**	**Modalidade da Linguagem**									
	**n(%)**	**Receptiva**			**Expressiva**			**Global**		
		**Média±DP**	**Mediana**	**p - valor**	**Média±DP**	**Mediana**	**p - valor**	**Média±DP**	**Mediana**	**p-valor**
**Via de parto**										
Vag.	10 (16,3)	27,60±16,81	27,00	0,191	26,80±19,74	28,00	0,339	54,40±36,47	55,00	0,246
Ces.	51 (83,6)	21,06±15,24	17,00		19,67±15,05	14,00		40,76±29,44	31,00	
**Hem. Intrac.**										
Sim	23 (37,7)	18,00±13,74	15,00	0,142	16,22±12,89	12,00	0,101	34,22±26,17	27,00	0,106
Não	38 (62,2)	24,63±16,22	25,00		23,63±17,10	18,00		48,32±32,44	42,00	

Teste U de Mann-Whitney

**Legenda:** n = número de indivíduos; DP = desvio padrão; vag = via de parto vaginal; ces. = via de parto cesária

**Tabela 4:** Associação entre fatores sociodemográficos e desenvolvimento linguístico

**Variáveis**	**Modalidade da Linguagem**									
		**Receptiva**			**Expressiva**			**Global**		
	**n(%)**	**Média±DP**	**Mediana**	**p -valor**	**Média±DP**	**Mediana**	**p - valor**	**Média±DP**	**Mediana**	**p-valor**
**Sexo**										
Masc.	37 (60,6)	19,68±15,26	15,00	0,112	18,05±14,33	12,00	0,148	37,78±28,73	28,00	0,140
Fe.	24 (39,3)	25,92±15,55	27,50		25,13±17,54	27,00		51,04±32,69	54,50	
**CCEB**										
A/B	9 (14,7)	26,67±15,62	31,00	0,642	25,00±14,92	28,00	0,756	51,89±29,50	55,00	0,740
C1	26 (42,6)	23,04±16,51	16,50		20,85±16,47	14,50		43,88±32,30	32,00	
C2	18 (29,5)	18,94±13,91	15,50		19,17±16,58	11,50		38,11±29,71	26,50	
D-E	8 (13,1)	21,25±17,19	24,50		19,88±16,03	21,50		41,13±32,98	47,00	

Teste U de Mann-Whitney

**Legenda:** n = número de participantes na amostra; DP = desvio padrão; Masc.= sexo masculino; Fe. = sexo feminino

**Tabela 5:** Correlação entre os fatores sociodemográficos, peri e pós-natais e desenvolvimento linguístico

**Variáveis**	**Linguagem Receptiva**	**Linguagem Expressiva**	**Linguagem Global**
Idade cronológica	0,888	0,839	0,890
Idade corrigida	0,236	0,535	0,545
Idade gestacional	0,056	0,035	0,035
Idade materna	0,020	0,041	0,031
Peso ao nascer	0,039	0,014	0,012
Estatura	0,143	0,159	0,154
Perímetro cefálico	0,109	0,066	0,088
Tempo de Internação	0,685	0,780	0,768
APGAR 1’	0,226	0,292	0,250
APGAR 5’	0,021	0,041	0,040

Coeficiente de Spearman

## DISCUSSÃO

O desenvolvimento linguístico de crianças prematuras é tema crucial na saúde infantil, especialmente considerando suas possíveis implicações a médio e longo prazo^(2,8)^. Fatores como sexo masculino, baixa classificação socioeconômica, via de parto cesáreo e ocorrência de hemorragia intracraniana que, frequentemente, são apontados como principais determinantes de um prognóstico restrito quanto ao desenvolvimento linguístico^(3)^, se mostraram influentes para um menor desempenho linguístico na amostra deste estudo.

Em pré-termos do sexo masculino há maiores riscos de alteração do neurodesenvolvimento a curto e a longo prazo, das habilidades cognitivas e motoras, além de ter maior tendência a apresentarem anormalidades no desenvolvimento de substância branca^(15)^. A distribuição da amostra mostrou que os escores obtidos nas modalidades receptiva, expressiva e global da linguagem revelou maior densidade de escores mais baixos no sexo masculino, o que corrobora estudos anteriores que indicam maior número de meninos com alterações no desenvolvimento da linguagem^(7,9)^. Vale destacar que no sexo feminino, houve uma distribuição simétrica dos escores obtidos, o que corrobora os resultados dos estudos mencionados^(7,9)^.

Um estudo que investigou os fatores de risco da prematuridade e sua relação com o desenvolvimento linguístico indicou a importância da avaliação nos primeiros 24 meses com idade corrigida^(7)^. Na amostra deste estudo, a idade corrigida foi considerada até 23 meses e 29 dias, representando quase um quarto de participantes. A idade cronológica, para análise, foi considerada a partir dos 24 meses de idade. Independente do cenário, este estudo não corrobora resultados anteriores, seja em estudos com crianças entre 0 e 24 meses de idade corrigida^(7,16)^ ou em estudos com idade cronológica igual ou superior a 24 meses^(9,16,17)^, que mostraram associação com significância estatística entre idade do prematuro e atraso do desenvolvimento linguístico.

O perfil clínico da prematuridade encontrado na amostra frequentemente está associado ao atraso de linguagem, conforme indicado na literatura^(7)^. Ao considerar a média da idade gestacional e o peso ao nascer obtidos, de acordo com os dados da Organização Mundial de Saúde, o perfil da amostra pode ser classificado como prematuridade severa e muito baixo peso ao nascer. A prematuridade extrema (IG <28 semanas) e os recém-nascidos muito baixo peso (RNMBP) foram associados a atrasos moderados e graves na linguagem^(7)^. No entanto, não foi encontrada diferença com significância estatística entre a idade gestacional de RNPT extremos e severos e os efeitos no desenvolvimento da linguagem^(18)^. Isso sugere que os riscos de alteração do desenvolvimento da linguagem são semelhantes ao considerar o nascimento antes das 32 semanas de idade gestacional.

No estudo, não houve associação com significância estatística entre as variáveis peso e idade gestacional e os escores de desenvolvimento linguístico, o que diverge dos resultados obtidos em outros estudos^(7,18)^. Nesta pesquisa, as variáveis linguísticas foram analisadas por meio de escores do instrumento utilizado, sem se valer de classificação de gravidade de alteração do desenvolvimento da linguagem, o que pode justificar o estudo não confirmar os achados prévios. A diversidade observada entre indivíduos prematuros, com diferentes pesos e idades gestacionais ao nascimento, sugere que estes grupos não são homogêneos em relação ao desenvolvimento. Isso é um desafio ao tentar obter uma noção de relação linear de causa e efeito entre prematuridade, peso ao nascer, idade gestacional e desenvolvimento linguístico. Portanto, ressalta-se a necessidade de investigar e identificar os mecanismos protetores que possam atenuar potenciais efeitos adversos do risco ao desenvolvimento de indivíduos prematuros^(17)^.

Estudos recentes divergem quanto à influência das medidas de perímetro cefálico e estatura no que concerne ao impacto no neurodesenvolvimento^(19,20,21)^ Enquanto um estudo observou que o perímetro cefálico apresentou desfecho desfavorável ao desenvolvimento neurocognitivo ao sexto mês de vida^(22)^, outro não achou relação entre essas variáveis^(20)^, assim como um estudo recente não achou relação entre estatura e neurodesenvolvimento^(21)^. O termo estatura é indiretamente relacionado às variáveis de peso ao nascer e idade gestacional, não sendo comum sua utilização em estudos prévios^(21)^.

A classe socioeconômica é uma variável de grande interesse dos pesquisadores, no que se refere aos fatores de risco para o desenvolvimento, não só da linguagem, mas de todo o aparato neuropsicomotor, em pré-termos. Algo importante a ser destacado é que, apesar de frequente a associação com significância estatística entre classes socioeconômicas e o desenvolvimento linguístico, há variabilidade considerável apresentada em diferentes estudos^(17,18)^. Uma revisão de literatura com metanálise evidenciou que cinco dos dezessete estudos analisados não apresentaram associação com significância estatística entre o desenvolvimento linguístico e classe socioeconômica^(17)^. Foi observado que mais de 40% das famílias das crianças avaliadas estavam classificadas na categoria C1, que infere uma renda média bruta de 2,2 salários mínimos por família^(14)^. Este percentual está acima da estimativa geral para o município onde está localizado o ambulatório de *follow-up*, que é de 23,4%, de acordo com o documento de validação e divulgação deste instrumento^(14)^. Nesta amostra, mais de dois terços das famílias foram classificadas nas classes C1 e C2 em que se observou menores escores nas modalidades receptiva, expressiva e global da linguagem, corroborando estudos que dizem que o contexto socioeconômico desfavorável está relacionado a menores desempenhos linguísticos^(7,12)^. Foi destacada anteriormente, a possibilidade de diferentes desfechos no desenvolvimento da linguagem ao considerar o fator socioeconômico^(17)^. Estudos recentes^(9,15)^ apresentam resultados similares, sugerindo que o sexo feminino, aliado a melhores condições socioeconômicas, resulta em um melhor desempenho em testes de linguagem. No contexto deste estudo, não foi obtida associação com significância estatística da classificação econômica com quaisquer variáveis dependentes. No entanto, é relevante salientar que os resultados obtidos fortalecem outros estudos que mostram uma influência forte do contexto socioeconômico no baixo desempenho linguístico.

A idade materna ao nascimento e a via de parto cesárea são fatores que influenciam o desfecho desfavorável ao prematuro^(22)^. Em um estudo de base populacional^(2)^ foi observado que a idade materna acima de 35 anos apresentou associação com significância estatística para atraso no desenvolvimento linguístico nos RNPT´s, enquanto Martins et al.^(3)^ mencionou em uma revisão de escopo que crianças prematuras de mães adolescentes apresentavam maior risco para o desenvolvimento cognitivo, o que incluí o desenvolvimento linguístico. A análise apresentada sugere que os extremos da idade fértil da mulher apresentam maiores associações com atraso do desenvolvimento linguístico. Em nossa amostra, a média obtida para a idade materna ao nascimento esteve entre os extremos apresentados, o que pode justificar a ausência de associação com significância estatística com o desenvolvimento linguístico.

Diante dos achados do estudo, foi possível observar que houve prevalência de partos por via cesárea, que corrobora um estudo que evidencia esta via de parto como prevalente em partos de crianças com histórico de prematuridade^(18)^. Mulheres que apresentam intercorrências na gestação apresentam maiores riscos de realizar cesáreas de emergência e promoverem o nascimento prematuro de seus bebês^(23)^, visto que a via de parto cesáreo, quando bem indicado, ajuda a reduzir riscos perinatais e aumentar a sobrevida dos recém-nascidos^(24)^. Na literatura foi descrita associação entre via de parto vaginal e melhor desempenho em testes de linguagem em prematuros^(3)^. Na análise dos dados obtidos neste estudo, não se observou associação com significância estatística entre essas duas variáveis. Contudo, foi possível observar que a via de parto cesárea mostrou maior densidade em escores mais baixos no sexo masculino em todas as modalidades da linguagem estudadas, o que indica que a via de parto vaginal e o sexo feminino predispõe melhor desempenho em testes que avaliam o desenvolvimento linguístico.

Em nossa amostra, a média dos valores na escala ao primeiro e ao quinto minuto sugere uma adaptação gradual ao ambiente extrauterino^(25)^. Em um estudo recente foi observado que os escores na escala APGAR ao primeiro minuto foi normal, ou seja, igual ou superior a 7, em prematuros nascidos com IG entre 28 e 37 semanas, não estabelecendo uma relação significativa com o desenvolvimento da linguagem^(8)^, assim como no presente estudo. Contudo, na literatura, também foi observado que escores de APGAR baixo no primeiro minuto esteve associado a distúrbio de linguagem^(23)^, enquanto outro estudo apontou significância estatística de menores escores de APGAR no primeiro e quinto minuto com distúrbio de linguagem^(26)^. A divergência entre os estudos indica a necessidade de investigação mais aprofundada sobre o impacto do escore APGAR no desenvolvimento linguístico.

A grande variabilidade do tempo de internação da amostra, demonstra que houve diferenças importantes entre os participantes. Vale considerar que a permanência prolongada do prematuro na UTIN é um risco para o neurodesenvolvimento^(8,25)^, pois restringe estímulos importantes para o início da maturação neuropsicomotora que se relaciona significativamente com desfechos desfavoráveis para cognição, linguagem e motricidade antes dos 12 meses de vida^(25)^.

A presença de hemorragia intracraniana (HIC) em pouco mais de um terço da amostra diverge de um estudo que revelou que essa condição não é tão prevalente em prematuros^(8)^. Entretanto, a ocorrência de hemorragia intracraniana em graus moderados e graves apresenta forte relação com atraso de linguagem^(7)^. No presente estudo não houve associação com significância estatística entre a ocorrência de hemorragia intracraniana com desenvolvimento linguístico, mas foi observado que no sexo feminino, as que tiveram HIC, os escores foram mais baixos, e as que não tiveram, observou-se o oposto. No sexo masculino, os escores de linguagem foram mais baixos independente da ocorrência ou não desta condição. Tais achados confirmam que a ocorrência da hemorragia intracraniana é um fator que predispõe atraso no desenvolvimento da linguagem, mas que outros fatores podem exercer maior influência sobre este desfecho clínico.

A discrepância no desenvolvimento das modalidades receptiva e expressiva da linguagem em crianças pré-termos foi apontada em estudos anteriores^(27)^. Estes estudos, embora considerassem escores de outros instrumentos de avaliação linguística, apontaram que, em prematuros, foi observada menor performance de habilidades linguísticas na modalidade expressiva em comparação à modalidade receptiva. Notavelmente, um desses estudos identificou diferença estatisticamente significativa entre os subtestes das duas modalidades linguísticas^(^ **^27^** ^)^. Na amostra do presente estudo, observou-se que na distribuição dos escores de ambas as modalidades, a média foi menor na modalidade expressiva do que na receptiva. Esse achado corrobora os estudos mencionados e faz jus às etapas de desenvolvimento linguístico, que prediz que a compreensão antecede a produção, sendo, portanto, o desenvolvimento de habilidades receptivas uma base pro desenvolvimento das habilidades expressivas da linguagem^(27)^.

Nenhuma das variáveis dependentes do estudo mostrou associação com significância estatística com quaisquer variáveis sociodemográficas ou fatores peri e pós-natais, apesar de terem sido observadas influências de algumas variáveis independentes nas variáveis de desenvolvimento linguístico. Como limitação do estudo, destaca-se o tamanho e delineamento amostral, o que impossibilita generalizar os resultados para outros contextos, além do serviço que foi cenário do estudo. O tamanho da amostra também impediu o agrupamento das crianças participantes em faixas etárias semelhantes, visto que na amostragem as idades foram heterogêneas. Portanto, é recomendado que novas pesquisas sejam realizadas com a utilização dessa ferramenta de avaliação linguística e que essas se dediquem às métricas padronizadas. É essencial ampliar a amostragem, buscando melhor distribuição entre as faixas etárias estudadas a fim de homogeneizar a análise dos resultados.

Em contrapartida, é importante ressaltar que este estudo contemplou a análise de diversas variáveis que foram apontadas anteriormente na literatura como influentes no desenvolvimento linguístico. Além disso, a amostra foi constituída por prematuros acompanhados em um serviço ambulatorial de *follow-up*, o que possibilitou o levantamento detalhado dos fatores de risco analisados. Outro ponto importante é que foi considerado o contexto socioeconômico das famílias, que vem sendo cada vez mais investigado por estudos correlatos, devido sua importante influência no espectro do desenvolvimento neuropsicomotor.

A avaliação a tempo e o acompanhamento longitudinal do desenvolvimento da linguagem têm por objetivo melhorar a qualidade de vida das crianças nascidas prematuras. Embora alguns resultados deste estudo não corroborem os achados de outras pesquisas^(5-11)^, que apontaram relações significativas entre o desenvolvimento da linguagem e as condições sociodemográficas, peri e pós-natais, são justificáveis e necessárias avaliações periódicas, padronizadas e criteriosas para identificar possíveis problemas no desenvolvimento infantil desde o início do desenvolvimento.

## CONCLUSÃO

Apesar da ausência de associações estatisticamente significativas entre os fatores sociodemográficos, peri e pós-natais e o desenvolvimento linguístico de prematuros acompanhados em um ambulatório de *follow-up*, os achados deste estudo preliminar apontam para tendências importantes. Observou-se uma maior concentração de escores mais baixos nas modalidades receptiva, expressiva e global da linguagem entre crianças do sexo masculino e pertencentes a estratos socioeconômicos mais baixos. Esses resultados, ainda que não conclusivos, reforçam a relevância de uma abordagem multidimensional na avaliação do desenvolvimento linguístico de prematuros, destacando a necessidade de estudos futuros com amostras maiores e delineamentos analíticos que possam aprofundar a compreensão dessas relações complexas. Por fim, é relevante enfatizar a importância do acompanhamento longitudinal, com avaliações periódicas do desenvolvimento linguístico, a fim de identificar precocemente possíveis alterações e propor intervenções adequadas para garantir o melhor aporte ao desenvolvimento e promover qualidade de vida aos prematuros a longo prazo.
